# Culture-Independent Analyses Reveal Novel Anaerolineaceae as Abundant Primary Fermenters in Anaerobic Digesters Treating Waste Activated Sludge

**DOI:** 10.3389/fmicb.2017.01134

**Published:** 2017-06-23

**Authors:** Simon J. McIlroy, Rasmus H. Kirkegaard, Morten S. Dueholm, Eustace Fernando, Søren M. Karst, Mads Albertsen, Per H. Nielsen

**Affiliations:** The Centre for Microbial Communities, Department of Chemistry and Bioscience, Aalborg UniversityAalborg, Denmark

**Keywords:** A6, anaerobic digestion, *Brevefilum fermentans*, Chloroflexi, FISH, metagenomics

## Abstract

Anaerobic digestion for biogas production is reliant on the tightly coupled synergistic activities of complex microbial consortia. Members of the uncultured A6 phylotype, within the phylum Chloroflexi, are among the most abundant genus-level-taxa of mesophilic anaerobic digester systems treating primary and surplus sludge from wastewater treatment plants, yet are known only by their 16S rRNA gene sequence. This study applied metagenomics to obtain a complete circular genome (2.57 Mbp) from a representative of the A6 taxon. Preliminary annotation of the genome indicates these organisms to be anaerobic chemoorganoheterotrophs with a fermentative metabolism. Given their observed abundance, they are likely important primary fermenters in digester systems. Application of fluorescence *in situ* hybridisation probes designed in this study revealed their morphology to be short filaments present within the flocs. The A6 were sometimes co-located with the filamentous Archaea *Methanosaeta* spp. suggesting potential undetermined synergistic relationships. Based on its genome sequence and morphology we propose the species name *Brevefilum fermentans* gen. nov. sp. nov.

## Introduction

Anaerobic digestion (AD) involves the conversion of organics to valuable methane, which is facilitated by the tightly coupled synergistic activities of complex microbial communities. The process essentially consists of four sequential microbial-mediated processes: hydrolysis, fermentation (acidogenesis), acetogenesis (dehydrogenation) and methanogenesis (acetoclastic or hydrogenotrophic) (Vanwonterghem et al., 2014). Members of the phylum Chloroflexi are widespread in full-scale ADs, constituting up to 50% of the bacterial community, and are largely confined to the family Anaerolineaceae (Nelson et al., 2011; Kirkegaard et al., 2017). Surprisingly, although their abundance indicates they must play a considerable role in these systems, their physiology and ecology is largely unknown. Most species of the Anaerolineaceae were isolated from anaerobic digester systems and have a fermentative metabolism, utilizing carbohydrates and proteinaceous carbon sources under anaerobic conditions (Sekiguchi et al., 2003; Yamada et al., 2006, 2007; Sun et al., 2016). A role in fermentation in AD systems is additionally supported by the annotation of available genomes derived from metagenomes (Xia et al., 2016) and with *in situ* evidence for the Chloroflexi phylum (Ariesyady et al., 2007). An in-depth understanding of the ecology and function of the Chloroflexi in biogas systems requires the characterisation of the abundant genera of the phylum.

A recent large scale amplicon sequencing survey of Danish full-scale AD communities revealed the A6 phylotype, a member of the Anaerolineaceae known only by their 16S rRNA gene sequence, to be among the most abundant genus-level-taxa in these systems; at times being present in excess of 20% of the amplicon reads (Kirkegaard et al., 2017). Advances in sequencing and metagenomic analyses enable the attainment of full genomes from the uncultured majority of microorganisms (Wrighton et al., 2012; Albertsen et al., 2013). In the absence of a pure culture, the aim of this study was to apply metagenomics to obtain a genome from a representative of the A6 phylotype, giving the first insight into their physiology.

## Materials and Methods

### Metagenome Preparation and Genome Binning

Metagenome sequencing and genome binning was performed essentially as detailed by Kirkegaard et al. (2016). Metagenomes were prepared from sludge obtained from the anaerobic digester tank at Fredericia wastewater treatment plant in Denmark. The Fredericia plant has 2 × 2000 m^3^ mesophilic digester tanks with Cambi^TM^ thermal high-pressure (THP) pre-treatment of influent sludge and treats 8000 metric tonnes dry sludge per year. Sludge was obtained from the digester at two different time points 36 weeks apart (March and December, 2011) enabling later differential coverage binning of genomes (Albertsen et al., 2013). The DNA was extracted from AD sludge using the FastDNA^®^ Spin kit for soil (MP Biomedicals, Santa Ana, CA, United States) following the standard protocol with minor modifications, as recommended by Albertsen et al. (2015). These differences included a four times increase in the duration of bead-beating and a sludge input volume of 50 μl.

Illumina TruSeq PCR free libraries and Nextera mate pair libraries were prepared according to the manufacturers’ protocol and paired-end sequenced (2 × 150 bp) on the Illumina HiSeq 2000 platform. The metagenomic reads were co-assembled using default settings in CLC Genomics Workbench (CLC Bio v. 7.5.1., Aarhus, Denmark). Reads were mapped to the assembly separately for each sample using default settings in CLC Genomics Workbench. The assembly and mapping information was exported as .fasta and .csv files, respectively, which were processed with the mmgenome workflow script ‘data.generation.2.1.0.sh’ to generate the files necessary for the binning process. Binning was carried out in the R environment using the mmgenome package^1^ (Karst et al., 2016). The raw metagenome reads and the annotated genome sequence data have been submitted to the European Nucleotide Archive (ENA) under the study accession number PRJEB19949.

### Genome Annotation

Genome annotation was performed in the ‘MicroScope’ annotation pipeline (Vallenet et al., 2013). Automatic annotations were validated manually for the genes involved in metabolic pathways of interest with the assistance of the integrated MicroCyc (Caspi et al., 2014) and KEGG (Kyoto Encyclopedia of Genes and Genomes) (Kanehisa et al., 2014) databases. The genome annotations are available on the ‘MicroScope’ website^2^.

### Probe Design and Fluorescence *In Situ* Hybridization (FISH)

Phylogenetic analysis and probe design was performed with the ARB software (Ludwig et al., 2004). Potential probes were assessed *in silico* with the mathFISH software (Yilmaz et al., 2011). The Ribosomal Database Project (RDP) PROBE MATCH function was used to screen for non-target sequences with single base indels (McIlroy et al., 2011; Cole et al., 2014). In order to improve the fluorescence *in situ* hybridization (FISH) signal to background ratio, both the 5′ and 3′ ends of oligonucleotide FISH probes were labeled with either the sulfoindocyanine dyes Cy3 or Cy5, or the 5(6)-carboxyfluorescein-*N*-hydroxysuccinimide ester (FLUOS) [DOPE-FISH (Stoecker et al., 2010)]. Probe validation and optimisation was based on generated formamide dissociation curves (Daims et al., 2005). With no pure cultures available, both probes were optimized using biomass with high abundance of the target organism from the anaerobic digester located at Ejby Mølle, Denmark (sampled August, 2013). In addition, CFX-A6-1278 was validated against *Pelolinea submarina* MO-CFX1^T^ which has a single base mismatch. Pure cultures with a single base mismatch to the CFX-A6-450 probe were not available. Probes designed in this study were deposited into the probeBase database (Greuter et al., 2016). The ARCH915 and the MX825mix (MX825; MX825b; MX825c) (Raskin et al., 1994; Crocetti et al., 2006) probes were applied to target the domain Archaea and the genus *Methanosaeta*, respectively. Quantitative FISH (qFISH) values were calculated as a percentage area of the total biovolume, stained with the 4′,6-diamidino-2-phenylindole (DAPI) DNA stain (50 μM, 1 h, at 4°C), which hybridized with the specific probe. The qFISH analyses were based on 25 fields of view taken at 630× magnification using the Daime image analyses software (DOME, Vienna, Austria) (Daims et al., 2006). Microscopy was performed with either an Axioskop epifluorescence microscope (Carl Zeiss, Oberkochen, Germany) or a White Light Laser Confocal Microscope (Leica TCS SP8 X) fitted with a 405 nm diode laser (Leica Microsystems, Kista, Sweden).

## Results and Discussion

**FIGURE 1:**
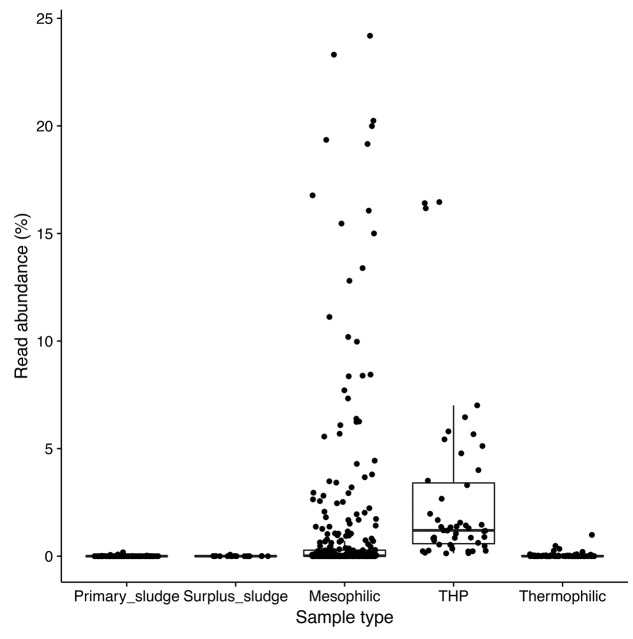
Box plot 16S rRNA gene amplicon sequence analysis (V1–3 region) of the distribution of the CAMBI-1 phylotype in full-scale ADs treating surplus sludge. Mesophilic AD – 15 plants, 321 samples; mesophilic AD with thermal high pressure (THP) pre-treatment of sludge (Cambi^TM^) – 2 plants, 47 samples; thermophilic – 5 plants, 102 samples; primary sludge – 14 plants, 121 samples; surplus sludge – 15 plants, 20 samples. Data is taken from the survey study of Kirkegaard et al. (2017) which the reader is referred to for further details.

**FIGURE 2:**
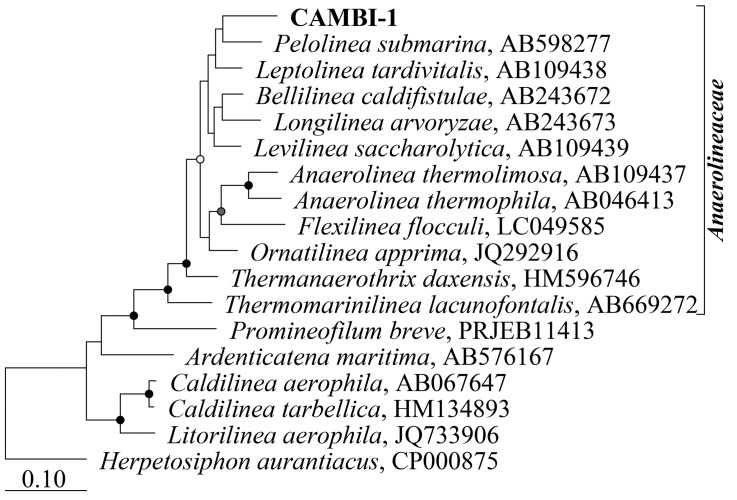
Maximum-likelihood (PhyML) 16S rRNA gene phylogenetic tree including CAMBI-1 and related described species within the phylum Chloroflexi. The tree was constructed using the ARB software with the SILVA SSU Ref NR99 v. 1.23 database (Quast et al., 2013). Additional sequences were aligned with the online SINA aligner with default settings (Pruesse et al., 2012) and imported into ARB. The alignment was trimmed and variable regions removed using a custom 40% base frequency filter giving 1372 aligned positions for tree calculation. *Herpetosiphon aurantiacus* was used to root the tree. Bootstrap values from 100 re-samplings are indicated for branches when >50%: white dots, >50%; gray, >70%; black, >90%. The scale bar represents substitutions per nucleotide base.

Amplicon sequencing survey data of full-scale ADs at wastewater treatment plants in Denmark showed a high abundance of the A6 phylotype in many of the mesophilic anaerobic digester tanks, but not in the primary or secondary sludge fed into these systems, suggesting that they are growing and well-adapted to mesophilic digester environment (**Figure 1**). In order to obtain genomes for the A6 taxon, metagenomes were generated for the Fredericia AD plant due to the observed high abundance of the target phylotype (representing up to 10% of the metagenome reads). A complete circular genome (CAMBI-1), classified to the novel MiDAS taxonomy defined A6 genus (McIlroy et al., 2017) based on its 16S rRNA gene sequence, was successfully assembled from the metagenomes (see **Table 1** for details). Phylogenetic analysis of the 16S rRNA gene revealed that CAMBI-1 clusters together with isolates of the Anaerolineaceae, sharing 85–90% 16S rRNA gene sequence identity (**Figure 2**). Based on the recommendations of Yarza et al. (2014), this indicates that CAMBI-1 should be considered to represent a novel genus within the family.

**Table 1:** Genome properties of the CAMBI-1 genome.

Property	
Size	2.57 Mbp
GC content	49.1%
Protein coding density	88.9%
CDS	2288
CDS assigned function^∗^	20.6%
rRNA operons	1
Sequencing project accession no.	PRJEB19949

*CDS, Coding DNA sequence; ^∗^MicroScope software prediction classes 1–3*.

**FIGURE 3:**
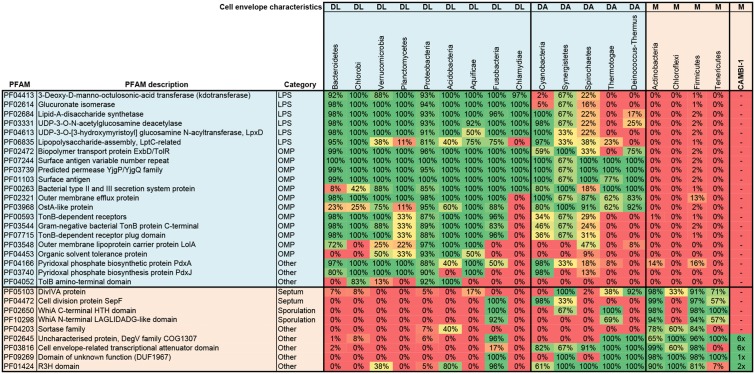
Cell envelope classification of CAMBI-1. Analysis was based on a search of the genome for genes encoding PFAM proteins (Finn et al., 2016) that are specific to archetypical mono- (M) or diderm bacteria with lipopolysaccharides (DL) or atypical diderm bacteria (DA) (as detailed previously by Albertsen et al., 2013). These include proteins involved in lipopolysaccharide synthesis (LPS), outer membrane associated proteins (OMP), and proteins associated with septum formation and sporulation. The percentage prevalence of each PFAM is given for each listed phylum. Phyla included are represented by all complete genomes (at least four each) in the IMG database (release 3.5) (Chen et al., 2017). The numbers shown in the column for CAMBI-1 represent the number of hits for a given PFAM protein in the analyzed genome. The PFAM profile of CAMBI-1 is similar to those of archetypical monoderm bacteria, including other members of the Chloroflexi.

Examination of the CAMBI-1 genome for PFAM proteins related to archetypic mono- and diderm cell envelopes, revealed a monoderm cell envelope architecture consistent with other Chloroflexi (**Figure 3**). The genome annotation and specialized searches using the PilFind program (Imam et al., 2011) did not reveal any genes associated with flagella, fimbriae or pili, suggesting a non-motile lifestyle. Putative genes associated with spore coat polysaccharide biosynthesis protein SpsC (CFX1CAM_0088; 1106) were annotated (Cangiano et al., 2014), although definitive candidates for other spore related genes were not found and their ability to form spore like structures is unclear.

**FIGURE 4:**
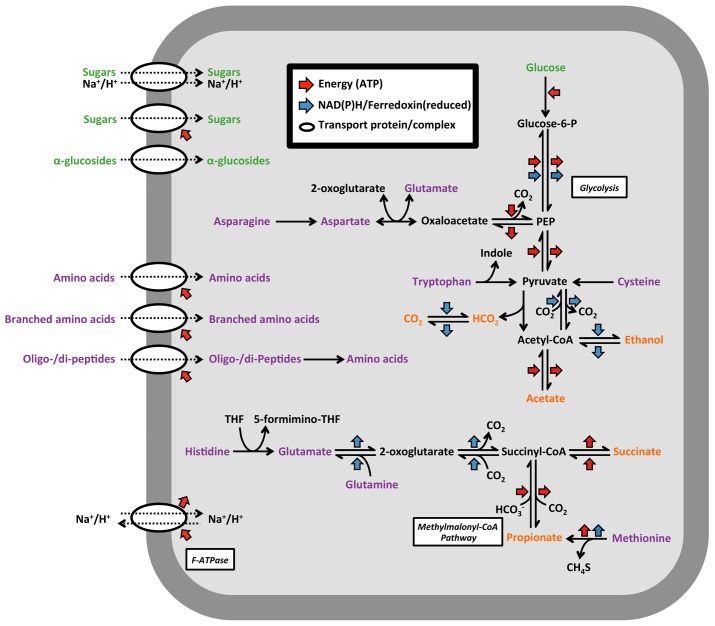
Selected catabolic pathways annotated in the A6 genome. Proteinaceous carbon substrates are given in purple, saccharide substrates in green and potential fermentation by-products in orange. PEP, phosphoenolpyruvate; THF, tetrahydrofolate.

The CAMBI-1 genome lacks a cytochrome oxidase, electron transport chain complexes and several key enzymes required for a complete TCA cycle, indicating a strict anaerobic metabolism. Annotation of a catalase (CFX1CAM_0578) and superoxide dismutase (CFX1CAM_2274) indicates some resistance to oxidative stress. Genes for the dissimilarity reduction of sulfate, nitrate or nitrite were also not annotated. Although an ability for denitrification was not annotated, the organism possesses a putative nitric oxide reductase (*norV*) (CFX1CAM_0414) and a putative hydroxylamine reductase (*hcp*) (CFX1CAM_0418), which both have suggested involvement in protection against nitrosative stress (Vine and Cole, 2011). Key genes for the Wood-Ljungdahl pathway and the Calvin-Benson-Bassham cycle were not annotated, indicating an inability to fix carbon for autotrophy. Potential for the pentose phosphate and Embden-Meyerhof-Parnas glycolysis pathways were present. Several annotated genes suggest a fermentative physiology consistent with other members of the family Anaerolineaceae (**Figure 4** and **Table 2**). Pyruvate can be converted to acetyl-CoA by a pyruvate: ferredoxin oxidoreductase (CFX1CAM_0326), pyruvate dehydrogenase (CFX1CAM_1724-1726) or a pyruvate formate lyase (CFX1CAM_0333), with formate released from activity of the latter potentially oxidized to CO_2_ by an annotated formate dehydrogenase (CFX1CAM_1212). Potential fermentation by-products from acetyl-CoA include acetate, mediated by an acetyl-CoA synthetase (CFX1CAM_0825; 1292), and ethanol, facilitated by acetaldehyde (CFX1CAM_1715) and alcohol dehydrogenases (CFX1CAM_0055). The annotation of putative genes associated with the methylmalonyl-CoA pathway (CFX1CAM_1019; 1020; 2064–2067) indicates that propionate could be produced as a metabolic by-product from the fermentation of amino acids. Annotated tungsten-containing aldehyde ferredoxin oxidoreductases (AORs) (CFX1CAM_1238; 2051) may function to oxidize aldehydes derived from amino acid oxidation (Heider et al., 1995). Several described members of the Anaerolineaceae (**Table 2**) produce hydrogen as a fermentation by-product. However, definitive evidence for a hydrogenase was not found in the CAMBI-1 genome.

**Table 2:** Summary of phenotypic characteristics of members of the family Anaerolineaceae.

Species	Isolation source	Temperature optimum	Physiology	Carbon sources/electron donors^∗^	Fermentation by-products (from sugars)	Reference
CAMBI-1	Anaerobic digester	Mesophile	Strict anaerobe; chemoheterotroph; fermenter	Carbohydrates; proteins^∗∗^	Acetate; ethanol; formate; CO_2_^∗∗^	This study
*Anaerolinea thermophila*^T^	Anaerobic digester	Thermophile	Strict anaerobe; chemoheterotroph; fermenter	Carbohydrates; proteins^w^	Acetate; H_2_; lactate^w^; succinate^w^; formate^w^	Sekiguchi et al., 2003
*Anaerolinea thermolimosa*^T^	Anaerobic digester	Thermophile	Strict anaerobe; chemoheterotroph; fermenter	Carbohydrates; proteins	Acetate; lactate; H_2_	Yamada et al., 2006
*Levilinea saccharolytica*^T^	Anaerobic digester	Mesophile	Strict anaerobe; chemoheterotroph; fermenter	Carbohydrates; proteins	Acetate; formate; H_2_; lactate^w^	Yamada et al., 2006
*Leptolinea tardivitalis*^T^	Anaerobic digester	Mesophile	Strict anaerobe; chemoheterotroph; fermenter	Carbohydrates; proteins	Acetate; lactate; pyruvate; H_2_; succinate^w^; formate^w^	Yamada et al., 2006
*Longilinea arvoryzae*^T^	Rice paddy soil	Mesophile	Strict anaerobe; chemoheterotroph; fermenter	Carbohydrates; proteins	Acetate; lactate; H_2_	Yamada et al., 2007
*Bellilinea caldifistulae*^T^	Anaerobic digester	Thermophile	Strict anaerobe; chemoheterotroph; fermenter	Carbohydrates; proteins^w^	Acetate; lactate; formate; H_2_; propionate^w^; pyruvate^w^	Yamada et al., 2007
‘Thermanaerothrix daxensis’^T^	Deep hot aquifier	Thermophile	Strict anaerobe; chemoheterotroph; fermenter	Carbohydrates	Lactate; acetate; CO_2_; H_2_^w^	Grégoire et al., 2011
*Thermomarinilinea lacunofontalis*^T^	Hydrothermal vent	Thermophile	Strict anaerobe; chemoheterotroph; fermenter	Proteins	–	Nunoura et al., 2013
*Ornatilinea apprima*^T^	Hot water bath microbial mat	Mesophile	Strict anaerobe; chemoheterotroph; fermenter	Carbohydrates; proteins	Acetate; ethanol; H_2_; lactate^w^; formate^w^	Podosokorskaya et al., 2013
*Pelolinea submarina*^T^	Marine sediment	Mesophile	Strict anaerobe; chemoheterotroph; fermenter	Carbohydrates	Acetate; lactate; ethanol; H_2_; pyruvate^w^; propionate^w^	Imachi et al., 2014
*Flexilinea flocculi*^T^	Anaerobic digester	Mesophile	Strict anaerobe; chemoheterotroph; fermenter	Carbohydrates	Acetate; lactate; succinate; propionate; formate; H_2_	Sun et al., 2016

*All listed species have a filamentous morphology; ^∗^ Proteins = protein based substrates rich in amino acids and peptides; ^∗∗^Not empirically demonstrated; ^w^Relatively weak growth observed/trace amounts produced*.

*^T^ Type species*.

**FIGURE 5:**
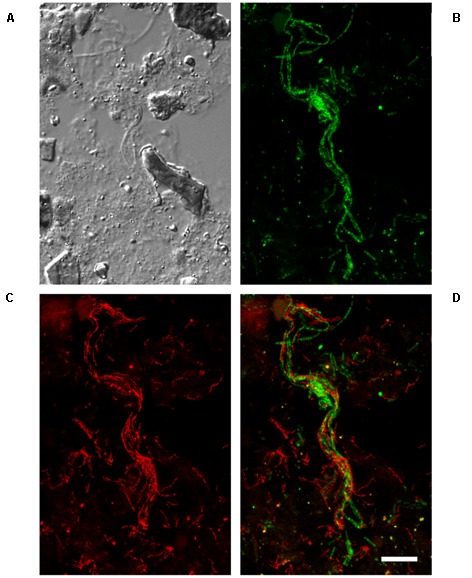
Fluorescence *in situ* hybridization (FISH) micrographs of the A6 in the Ejby Mølle AD, Denmark (sampled February, 2013). **(A)** DIC image of the biomass. **(B)** FISH image with MX825mix probe set (MX825 + MX825b + MX825c) targeting the genus *Methanosaeta* (green). **(C)** FISH image with the CFX-A6-mix probe set (CFX-A6-1278 + CFX-A6-450: red). **(D)** Overlay image of CFX-A6-mix and MX825mix images showing co-location of the A6 (red filaments) and *Methanosaeta* spp. (green filaments). All images are from the same field of view. Scale bar = 10 μm.

Fluorescence *in situ* hybridisation probes were designed to visualize the morphology of the A6 *in situ* (**Table 3**). The CFX-A6-450 and CFX-A6-1278 probes were designed to cover the phylotype. These can be applied together with different fluorochromes, where the overlap gives a higher confidence in specificity, or with the same fluorochrome to give a higher coverage of the group and to increase the signal to background ratio that can be problematic with AD samples. Application of these probes to the Fredericia AD biomass, and several additional full-scale digesters, revealed that these organisms form short filaments that are typically approximately 0.3 μm thick and 5–10 μm long (**Figure 5C**), but were occasionally observed at lengths of >100 μm. Unlabelled helper probes were designed for the CFX-A6-1278 and CFX-A6-450 probes, but only CFX-A6-1278_H1 gave an increase in fluorescence and is recommended for use (**Table 3**). Competitor probes were designed to cover un-validated single base mismatches in non-target sequences (**Table 3**). Stringency of the CFX-A6-1278 probe was supported by its application to *P. submarina* MO-CFX1^T^ – a non-target isolate with a single mismatch to the probe – which gave no positive fluorescent signal. Unlike some prominent wastewater-related Chloroflexi (Kragelund et al., 2007, 2011; Speirs et al., 2009), the A6 are covered by the EUBmix FISH probe set routinely applied to cover most members of the domain bacteria (Amann et al., 1990; Daims et al., 1999).

**Table 3:** FISH probes designed in this study.

Probe	*E. coli* pos.	Target group	*Coverage*^∗^	Sequence (5′-3′)	[FA]%^∗∗^
CFX-A6-1278	1278–1298	A6 clade	81%	GAG GCC TGC TTT CAG GAT TG	45
CFX-A6-1278_C1	1278–1298	Competitor probe for CFX-A6-1278	N/A	GAG GCC GGC TTT CAG GAT TG	–
CFX-A6-1278_C2^∗∗∗^	1278–1298	Competitor probe for CFX-A6-1278	N/A	GAG GCC TGC TTT DAG GAT TG	–
CFX-A6-1278_H1	1262–1277	Helper probe for CFX-A6-1278	N/A	GCT CCG CCT YGC GRC T	–
CFX-A6-1278_H2^∗∗∗∗^	1299–1322	Helper probe for CFX-A6-1278	N/A	GRG TTG CAG ACT GCA ATC TGA ACT	–
CFX-A6-450	450–492	A6 clade	86%	GGG AGT ACA GTC CTT CCT C	40
CFX-A6-450_C	450–492	Competitor probe for CFX-A6-450	N/A	GGG AGT ACY GTC CTT CCT C	–
CFX-A6-450_H^∗∗∗∗^	494–519	Helper probe for CFX-A6-450	N/A	GGC ACG TAG TTA GCC GAG ACT TAT TC	–
CFX-A6-mix	N/A	A6 clade	96%	CFX-A6-1278 + CFX-A6-450	45

*^∗^Coverage based on the MiDAS taxonomy version 2.1 (McIlroy et al., 2017). There were no non-target hits for either probe. ^∗∗^Optimal hybridisation formamide concentration % [v/v]. ^∗∗∗^The CFX-A6-1278 probe did not give a positive signal when applied to *Pelolinea submarina* MO-CFX1^*T*^ (at the recommended formamide concentration), which represents the non-target single base mismatched sequences covered by this competitor probe – the CFX-A6-1278_C2 probe is therefore not required. ^∗∗∗∗^Addition of these helper probes did not improve fluorescence when applied with their respective probes and are therefore not required*.

The A6 were distributed throughout the flocs and were sometimes observed co-located with the filamentous Archaea *Methanosaeta* spp. (**Figure 5**), indicating the potential for synergistic relationships. Parallel growth of these two filamentous genera was observed in multiple plants but was only frequent in the Ejby Mølle AD (23% of *Methanosaeta* filaments having associated A6; see **Table 4**). Unfortunately, the low FISH signal to background ratio for the thin A6 filaments did not permit qFISH studies or statistical co-location analyses (Daims et al., 2006); noting that visual assessment often indicated a higher abundance of A6 relative to the *Methanosaeta* spp., with most of both genera growing independent of contact with the other. Amplicon sequencing surveys indicate that the *Methanosaeta* are the most abundant methanogenic archaeal genus in mesophilic ADs located at WWTPs in Denmark (Kirkegaard et al., 2017), which was supported by qFISH in this study where they made up approximately half of the archaeal biovolume (**Table 4**). As such, the potential synergistic relationship with the A6 filaments may have important implications for methane production in some full-scale AD systems (e.g., Ejby Mølle). *Methanosaeta* are widely known to be acetoclastic methanogens (Whitman et al., 2014), which would therefore benefit from the use of any acetate theoretically produced by the fermentative A6. It has also been demonstrated that some *Methanosaeta* species in ADs can utilize electrons sourced through direct interspecies electron transfer (DIET), from ethanol oxidizing *Geobacter* spp., to reduce CO_2_ to methane (Rotaru et al., 2014). Thus, the A6 may transfer excess electrons from the oxidation of organic carbon to the *Methanosaeta* via DIET, facilitated by the observed close proximity of the two organisms. It is thought that cytochromes and electrically conductive pili structures facilitate electrons flow to the recipient organism (Shrestha and Rotaru, 2014). A single membrane associated polyheme cytochrome c (CFX1CAM_1800) was annotated which had low homology to known DIET cytochromes (22% amino acid sequence similarity with OmcS from *Geobacter sulfurreducens* (Acc. No. AAR35877)), though no pili associated genes were identified in the CAMBI-1 genome. It may be that novel conductive structures are utilized by these organisms, or the CAMBI-1 genome may not represent the A6 species co-located with the *Methanosaeta*. Further work into the possibility for DIET and other potential interactions between the two genera is required.

**Table 4:** FISH analyses of the abundance of the *Methanosaeta* spp. and their association with the A6.

Measurement	AD location			
	Ejby Mølle	Slagelse	Randers	Aalborg East
Archaea % biovolume^∗^	4 ± 2	2 ± 1	5 ± 1	2 ± 1
*Methanosaeta* % biovolume^∗^	2 ± 1	1 ± 1	3 ± 1	1 ± 1
% *Methanosaeta* with associated A6^∗∗^	23 ± 3	2 ± 1	1 ± 1	0

*Values are averages ± standard deviation, calculated from ^∗^25 image sets or ^∗∗^3 manual counts of 100 randomly selected *Methanosaeta* filaments (>10 μm in length). *Methanosaeta* filaments were considered positive for association with A6 filaments if multiple of the latter appeared horizontally attached to the former. 12 other mesophilic ADs located in Denmark were screened but excluded from analyses due to high background fluorescence or the absence or low abundance of the A6*.

This study provides the first insight into the ecology of the A6 phylotype. Genomic evidence, coupled with their high relative abundance, suggests that members of the phylotype are important fermenters in mesophilic AD systems. The annotation of the representative CAMBI-1 genome gives a basic understanding of their physiology, noting that most of the coding sequence was not associated with any function (**Table 1**). A detailed understanding of the ecology of the A6 will be achieved with *in situ* and gene expression studies and by obtaining axenic cultures for representatives of the genus. The attainment of a genome and the optimisation of FISH probes in this study provides an important foundation for these approaches. Importantly, having complete genomes representing the abundant members of the community is an essential reference for metatranscriptomic and metaproteomic gene expression studies that will together allow organism-based metabolic networks to be developed for anaerobic digester systems – giving a comprehensive view of the ecology of these biotechnologically important systems (Waldor et al., 2015).

## Taxonomic Proposal

In the absence of a pure culture, and with only partial characterisation, organisms have previously been given provisional Candidatus status names (Murray and Stackebrandt, 1995). However, in accordance with the recent recommendations of Whitman (2016), regarding the naming of uncultured organisms where the genome sequence is known, we propose the name *Brevefilum fermentans* gen. nov. sp. nov. with the CAMBI-1 genome as a provisional type species reference.

Bre’veh.fi.lum fer.men’tans. L. adj. *brevis* short; L. neutral. n. *filum* line; L. part. adj. *fermentans* fermenting. *Brevefilum fermentans* a fermenting short filament.

## Author Contributions

SM, RK, and PN planned the experimental work. SM and EF performed the FISH-based analyses. RK performed the DNA-based experimental work. RK, SK, and MA performed the metagenomic analyses. SM and MD performed the genome annotation. The manuscript was written by SM and drafted by all authors.

## Conflict of Interest Statement

The authors declare that the research was conducted in the absence of any commercial or financial relationships that could be construed as a potential conflict of interest.

## Footnotes

1 https://github.com/MadsAlbertsen/mmgenome

2 http://www.genoscope.cns.fr/agc/microscope/home/

## References

[B1] Albertsen M. Hugenholtz P. Skarshewski A. Nielsen K. L. Tyson G. W. Nielsen P. H. (2013). Genome sequences of rare, uncultured bacteria obtained by differential coverage binning of multiple metagenomes. *Nat. Biotechnol.* 31 533–538. 10.1038/nbt.2579 23707974

[B2] Albertsen M. Karst S. M. Ziegler A. S. Kirkegaard R. H. Nielsen P. H. (2015). Back to basics – the influence of DNA extraction and primer choice on phylogenetic analysis of activated sludge communities. *PLoS ONE* 10:e0132783 10.1371/journal.pone.0132783 PMC4504704 26182345

[B3] Amann R. I. Binder B. J. Olson R. J. Chisolm S. W. Devereux R. Stahl D. A. (1990). Combination of 16S rRNA-targeted oligonucleotide probes with flow cytometry for analyzing mixed microbial populations. *Appl. Environ. Microbiol.* 56 1919–1925. 2200342 10.1128/aem.56.6.1919-1925.1990 PMC184531

[B4] Ariesyady H. D. Ito T. Okabe S. (2007). Functional bacterial and archaeal community structures of major trophic groups in a full-scale anaerobic sludge digester. *Water Res.* 41 1554–1568. 10.1016/j.watres.2006.12.036 17291558

[B5] Cangiano G. Sirec T. Panarella C. Isticato R. Baccigalupi L. De Felice M. (2014). The sps gene products affect the germination, hydrophobicity, and protein adsorption of *Bacillus subtilis* spores. *Appl. Environ. Microbiol.* 80 7293–7302. 10.1128/AEM.02893-14 25239894 PMC4249184

[B6] Caspi R. Altman T. Billington R. Dreher K. Foerster H. Fulcher C. A. (2014). The MetaCyc database of metabolic pathways and enzymes and the BioCyc collection of pathway/genome databases. *Nucleic Acids Res.* 42 D459–D471. 10.1093/nar/gkt1103 24225315 PMC3964957

[B7] Chen I. A. Markowitz V. M. Chu K. Palaniappan K. Szeto E. Pillay M. (2017). IMG/M: integrated genome and metagenome comparative data analysis system. *Nucleic Acids Res.* 45 D507–D516. 10.1093/nar/gkw929 27738135 PMC5210632

[B8] Cole J. R. Wang Q. Fish J. A. Chai B. McGarrell D. M. Sun Y. (2014). Ribosomal database project: data and tools for high throughput rRNA analysis. *Nucleic Acids Res.* 42 D633–D642. 10.1093/nar/gkt1244 24288368 PMC3965039

[B9] Crocetti G. Murto M. Björnsson L. (2006). An update and optimisation of oligonucleotide probes targeting methanogenic *Archaea* for use in fluorescence in situ hybridisation (FISH). *J. Microbiol. Methods* 65 194–201. 10.1016/j.mimet.2005.07.007 16126291

[B10] Daims H. Brühl A. Amann R. Schleifer K. H. Wagner M. (1999). The domain-specific probe EUB338 is insufficient for the detection of all Bacteria: development and evaluation of a more comprehensive probe set. *Syst. Appl. Microbiol.* 22 434–444. 10.1016/S0723-2020(99)80053-8 10553296

[B11] Daims H. Lücker S. Wagner M. (2006). daime, a novel image analysis program for microbial ecology and biofilm research. *Environ. Microbiol.* 8 200–213. 10.1111/j.1462-2920.2005.00880.x 16423009

[B12] Daims H. Stoecker K. Wagner M. (2005). “Fluorescence *in situ* hybridization for the detection of prokaryotes,” in *Molecular Microbial Ecology*, eds Osborn A. M. Smith C. J. (New York, NY: Taylor & Francis), 213–239.

[B13] Finn R. D. Coggill P. Eberhardt R. Y. Eddy S. R. Mistry J. Mitchell A. L. (2016). The Pfam protein families database: towards a more sustainable future. *Nucleic Acids Res.* 44 D279–D285. 10.1093/nar/gkv1344 26673716 PMC4702930

[B14] Grégoire P. Fardeau M.-L. Joseph M. Guasco S. Hamaide F. Biasutti S. (2011). Isolation and characterization of *Thermanaerothrix daxensis* gen. nov., sp. nov., a thermophilic anaerobic bacterium pertaining to the phylum “*Chloroflexi*”, isolated from a deep hot aquifer in the Aquitaine Basin. *Syst. Appl. Microbiol.* 34 494–497. 10.1016/j.syapm.2011.02.004 21621938

[B15] Greuter D. Loy A. Horn M. Rattei T. (2016). probeBase - an online resource for rRNA-targeted oligonucleotide probes and primers: new features 2016. *Nucleic Acids Res.* 44 D586–D589. 10.1093/nar/gkv1232 26586809 PMC4702872

[B16] Heider J. Ma K. Adams M. (1995). Purification, characterization, and metabolic function of tungsten- containing aldehyde ferredoxin oxidoreductase from the hyperthermophilic and proteolytic archaeon *Thermococcus* strain ES-1. *J. Bacteriol.* 177 4757–4764. 7642503 10.1128/jb.177.16.4757-4764.1995 PMC177242

[B17] Imachi H. Sakai S. Lipp J. S. Miyazaki M. Saito Y. Yamanaka Y. (2014). *Pelolinea submarina* gen. nov., sp. nov., an anaerobic, filamentous bacterium of the phylum *Chloroflexi* isolated from subseafloor sediment. *Int. J. Syst. Evol. Microbiol.* 64 812–818. 10.1099/ijs.0.057547-0 24215824

[B18] Imam S. Chen Z. Roos D. S. Pohlschröder M. (2011). Identification of surprisingly diverse type IV pili, across a broad range of Gram-positive bacteria. *PLoS ONE* 6:e28919 10.1371/journal.pone.0028919 PMC3244431 22216142

[B19] Kanehisa M. Goto S. Sato Y. Kawashima M. Furumichi M. Tanabe M. (2014). Data, information, knowledge and principle: back to metabolism in KEGG. *Nucleic Acids Res.* 42 D199–D205. 10.1093/nar/gkt1076 24214961 PMC3965122

[B20] Karst S. M. Kirkegaard R. H. Albertsen M. (2016). mmgenome: a toolbox for reproducible genome extraction from metagenomes. *bioRxiv* 10.1101/059121

[B21] Kirkegaard R. H. Dueholm M. S. McIlroy S. J. Nierychlo M. Karst S. M. Albertsen M. (2016). Genomic insights into members of the Candidate phylum Hyd24-12 common in mesophilic anaerobic digesters. *ISME J.* 10 2352–2364. 10.1038/ismej.2016.43 27058503 PMC5030696

[B22] Kirkegaard R. H. McIlroy S. J. Kristensen J. M. Nierychlo M. Karst S. M. Dueholm M. S. (2017). Identifying the abundant and active microorganisms common to full scale anaerobic digesters. *bioRxiv* 10.1101/104620

[B23] Kragelund C. Levantesi C. Borger A. Thelen K. Eikelboom D. Tandoi V. (2007). Identity, abundance and ecophysiology of filamentous *Chloroflexi* species present in activated sludge treatment plants. *FEMS Microbiol. Ecol.* 59 671–682. 10.1111/j.1574-6941.2006.00251.x 17381520

[B24] Kragelund C. Thomsen T. R. Mielczarek A. T. Nielsen P. H. (2011). Eikelboom’s morphotype 0803 in activated sludge belongs to the genus *Caldilinea* in the phylum *Chloroflexi*. *FEMS Microbiol. Ecol.* 76 451–462. 10.1111/j.1574-6941.2011.01065.x 21299573

[B25] Ludwig W. Strunk O. Westram R. Richter L. Meier H. Yadhukumar (2004). ARB: a software environment for sequence data. *Nucleic Acids Res.* 32 1363–1371. 10.1093/nar/gkh293 14985472 PMC390282

[B26] McIlroy S. J. Kirkegaard R. H. McIlroy B. Nierychlo M. Kristensen J. M. Karst S. M. (2017). MiDAS 2.0: an ecosystem-specific taxonomy and online database for the organisms of wastewater treatment systems expanded for anaerobic digester groups. *Database* 2017:bax016 10.1093/database/bax016 PMC5467571 28365734

[B27] McIlroy S. J. Tillett D. Petrovski S. Seviour R. J. (2011). Non-target sites with single nucleotide insertions or deletions are frequently found in 16S rRNA sequences and can lead to false positives in fluorescence *in situ* hybridization (FISH). *Environ. Microbiol.* 13 38–47. 10.1111/j.1462-2920.2010.02306.x 20649647

[B28] Murray R. G. Stackebrandt E. (1995). Taxonomic note: implementation of the provisional status Candidatus for incompletely described procaryotes. *Int. J. Syst. Bacteriol.* 45 186–187. 7857801 10.1099/00207713-45-1-186

[B29] Nelson M. C. Morrison M. Yu Z. (2011). A meta-analysis of the microbial diversity observed in anaerobic digesters. *Bioresour. Technol.* 102 3730–3739. 10.1016/j.biortech.2010.11.119 21194932

[B30] Nunoura T. Hirai M. Miyazaki M. Kazama H. Makita H. Hirayama H. (2013). Isolation and characterization of a thermophilic, obligately anaerobic and heterotrophic marine *Chloroflexi* bacterium from a *Chloroflexi* dominated microbial community associated with a japanese shallow hydrothermal system, and proposal for *Thermomarinilinea lacunofontalis* gen. nov., sp. nov. *Microbes Environ.* 28 228–235. 10.1264/jsme2.ME12193 23666537 PMC4070665

[B31] Podosokorskaya O. A. Bonch-Osmolovskaya E. A. Novikov A. A. Kolganova T. V. Kublanov I. V. (2013). *Ornatilinea apprima* gen. nov., sp. nov., a cellulolytic representative of the class *Anaerolineae*. *Int. J. Syst. Evol. Microbiol.* 63 86–92. 10.1099/ijs.0.041012-0 22328612

[B32] Pruesse E. Peplies J. Glöckner F. O. (2012). SINA: accurate high-throughput multiple sequence alignment of ribosomal RNA genes. *Bioinformatics* 28 1823–1829. 10.1093/bioinformatics/bts252 22556368 PMC3389763

[B33] Quast C. Pruesse E. Yilmaz P. Gerken J. Schweer T. Yarza P. (2013). The SILVA ribosomal RNA gene database project: improved data processing and web-based tools. *Nucleic Acids Res.* 41 D590–D596. 10.1093/nar/gks1219 23193283 PMC3531112

[B34] Raskin L. Stromley J. M. Rittmann B. E. Stahl D. A. (1994). Group-Specific 16S rRNA hybridization probes to describe natural communities of methanogens. *Appl. Environ. Microbiol.* 60 1232–1240. 7517128 10.1128/aem.60.4.1232-1240.1994 PMC201464

[B35] Rotaru A.-E. Shrestha P. M. Liu F. Shrestha M. Shrestha D. Embree M. (2014). A new model for electron flow during anaerobic digestion: direct interspecies electron transfer to *Methanosaeta* for the reduction of carbon dioxide to methane. *Energy Environ. Sci.* 7 408–415. 10.1039/c3ee42189a

[B36] Sekiguchi Y. Yamada T. Hanada S. Ohashi A. Harada H. Kamagata Y. (2003). *Anaerolinea thermophila* gen. nov., sp. nov. and *Caldilinea aerophila* gen. nov., sp. nov., novel filamentous thermophiles that represent a previously uncultured lineage of the domain *Bacteria* at the subphylum level. *Int. J. Syst. Evol. Microbiol.* 53 1843–1851. 10.1099/ijs.0.02699-0 14657113

[B37] Shrestha P. M. Rotaru A.-E. (2014). Plugging in or going wireless: strategies for interspecies electron transfer. *Front. Microbiol.* 5:237 10.3389/fmicb.2014.00237 PMC4032928 24904551

[B38] Speirs L. Nittami T. McIlroy S. Schroeder S. Seviour R. J. (2009). Filamentous bacterium Eikelboom type 0092 in activated sludge plants in Australia is a member of the phylum *Chloroflexi*. *Appl. Environ. Microbiol.* 75 2446–2452. 10.1128/AEM.02310-08 19218415 PMC2675213

[B39] Stoecker K. Dorninger C. Daims H. Wagner M. (2010). Double labeling of oligonucleotide probes for fluorescence in situ hybridization (DOPE-FISH) improves signal intensity and increases rRNA accessibility. *Appl. Environ. Microbiol.* 76 922–926. 10.1128/AEM.02456-09 19966029 PMC2813028

[B40] Sun L. Toyonaga M. Ohashi A. Matsuura N. Tourlousse D. M. Meng X.-Y. (2016). Isolation and characterization of *Flexilinea flocculi* gen. nov., sp. nov., a filamentous anaerobic bacterium belonging to the class *Anaerolineae* in the phylum *Chloroflexi*. *Int. J. Syst. Evol. Microbiol.* 66 988–996. 10.1099/ijsem.0.000822 26637817

[B41] Vallenet D. Belda E. Calteau A. Cruveiller S. Engelen S. Lajus A. (2013). MicroScope–an integrated microbial resource for the curation and comparative analysis of genomic and metabolic data. *Nucleic Acids Res.* 41 D636–D647. 10.1093/nar/gks1194 23193269 PMC3531135

[B42] Vanwonterghem I. Jensen P. D. Ho D. P. Batstone D. J. Tyson G. W. (2014). Linking microbial community structure, interactions and function in anaerobic digesters using new molecular techniques. *Curr. Opin. Biotechnol.* 27 55–64. 10.1016/j.copbio.2013.11.004 24863897

[B43] Vine C. E. Cole J. A. (2011). Unresolved sources, sinks, and pathways for the recovery of enteric bacteria from nitrosative stress. *FEMS Microbiol. Lett.* 325 99–107. 10.1111/j.1574-6968.2011.02425.x 22029434

[B44] Waldor M. K. Tyson G. Borenstein E. Ochman H. Moeller A. Finlay B. B. (2015). Where next for microbiome research? *PLoS Biol.* 13:e1002050 10.1371/journal.pbio.1002050 PMC4300079 25602283

[B45] Whitman W. B. (2016). Modest proposals to expand the type material for naming of prokaryotes. *Int. J. Syst. Evol. Microbiol.* 66 2108–2112. 10.1099/ijsem.0.000980 26902077

[B46] Whitman W. B. Bowen T. L. Boone D. R. (2014). “The methanogenic bacteria,” in *The Prokaryotes*, eds Rosenberg E. Long E. De Lory S. Stackebrandt E. Thomson F. (Berlin: Springer), 123–163. 10.1007/978-3-642-38954-2_407

[B47] Wrighton K. C. Thomas B. C. Sharon I. Miller C. S. Castelle C. J. VerBerkmoes N. C. (2012). Fermentation, hydrogen, and sulfur metabolism in multiple uncultivated bacterial phyla. *Science* 337 1661–1665. 10.1126/science.1224041 23019650

[B48] Xia Y. Wang Y. Wang Y. Chin F. Y. L. Zhang T. (2016). Cellular adhesiveness and cellulolytic capacity in *Anaerolineae* revealed by omics-based genome interpretation. *Biotechnol. Biofuels* 9:111 10.1186/s13068-016-0524-z PMC4877987 27222666

[B49] Yamada T. Imachi H. Ohashi A. Harada H. Hanada S. Kamagata Y. (2007). *Bellilinea caldifistulae* gen. nov., sp. nov. and *Longilinea arvoryzae* gen. nov., sp. nov., strictly anaerobic, filamentous bacteria of the phylum *Chloroflexi* isolated from methanogenic propionate-degrading consortia. *Int. J. Syst. Evol. Microbiol.* 57 2299–2306. 10.1099/ijs.0.65098-0 17911301

[B50] Yamada T. Sekiguchi Y. Hanada S. Imachi H. Ohashi A. Harada H. (2006). *Anaerolinea thermolimosa* sp. nov., *Levilinea saccharolytica* gen. nov., sp. nov. and *Leptolinea tardivitalis* gen. nov., sp. nov., novel filamentous anaerobes, and description of the new classes *Anaerolineae* classis nov. and *Caldilineae* classis nov. in the bacterial phylum *Chloroflexi*. *Int. J. Syst. Evol. Microbiol.* 56 1331–1340. 10.1099/ijs.0.64169-0 16738111

[B51] Yarza P. Yilmaz P. Pruesse E. Glöckner F. O. Ludwig W. Schleifer K.-H. (2014). Uniting the classification of cultured and uncultured bacteria and archaea using 16S rRNA gene sequences. *Nat. Rev. Microbiol.* 12 635–645. 10.1038/nrmicro3330 25118885

[B52] Yilmaz L. S. Parnerkar S. Noguera D. R. (2011). mathFISH, a web tool that uses thermodynamics-based mathematical models for *in silico* evaluation of oligonucleotide probes for fluorescence *in situ* hybridization. *Appl. Environ. Microbiol.* 77 1118–1122. 10.1128/AEM.01733-10 21148691 PMC3028703

